# Long Non-Coding RNA-Mediated Regulation of the Interferon Response: A New Perspective on a Familiar Theme

**DOI:** 10.20411/pai.v3i1.252

**Published:** 2018-08-10

**Authors:** Saba Valadkhan, Leah M. Plasek

**Affiliations:** 1 Department of Molecular Biology and Microbiology, Case Western Reserve University School of Medicine, Cleveland, Ohio

**Keywords:** Long Non-Coding RNA, interferon response, natural killer cells

## Abstract

The interferon (IFN) response is a critical and ubiquitous component of the innate immune response to pathogens. Detailed studies in the last decades have elucidated the function of a large number of proteins that mediate the complex signaling pathways and gene expression programs involved in the interferon response. The recent discovery of the long non-coding RNAs (lncRNAs) as a new category of cellular effectors has led to studies aiming to understand the role of these transcripts in the IFN response. Several high throughput studies have shown that a large number of lncRNAs are differentially expressed following IFN stimulation and/or viral infections. In-depth study of a very small fraction of the identified lncRNAs has revealed critical roles for this class of transcripts in the regulation of multiple steps of the IFN response, and pointed to the presence of an extensive RNA-mediated regulatory network during the antiviral response. As the vast majority of the identified potential regulatory lncRNAs remain unstudied, it is highly likely that future studies will reveal a completely new perspective on the regulation of the IFN response, with lncRNA- and protein-mediated regulatory networks coordinating the duration, magnitude, and character of this aspect of the innate immune response. In addition to providing a more complete picture of the IFN response, these studies will likely identify new therapeutic targets that in the long term may impact the therapeutic options available against microbial infections and diseases of the immune system.

A critical part of the innate immune response against viruses and a wide variety of other microbial pathogens is mediated by the interferon (IFN) response, a ubiquitous and powerful defense mechanism aiming to limit the replication of the invading organism and help initiate a concerted immune response. The first step in the initiation of the IFN response is the recognition of the pathogen-associated molecular patterns. This leads to the induction of the expression of IFN genes, which in turn result in activation of extensive transcriptional cascades culminating in expression of antiviral and antibacterial genes. To control and optimize this powerful remodeling of the cellular transcriptome and proteome, the IFN response also triggers an intricate and poorly understood regulatory network. Through integration of intra- and extracellular signals from diverse origins, this regulatory mechanism serves to adjust the magnitude, duration, and character of the IFN response. In addition, IFNs regulate other components of the innate and adaptive immune response such as natural killer (NK) cell function, B cell antibody production, and the effector function of T cells, and thus help in coordinating the organismal defense against micro-organisms [1].

## INITIATION OF THE IFN RESPONSE AND INDUCTION OF EXPRESSION OF THE IFN GENES

**Figure 1.**
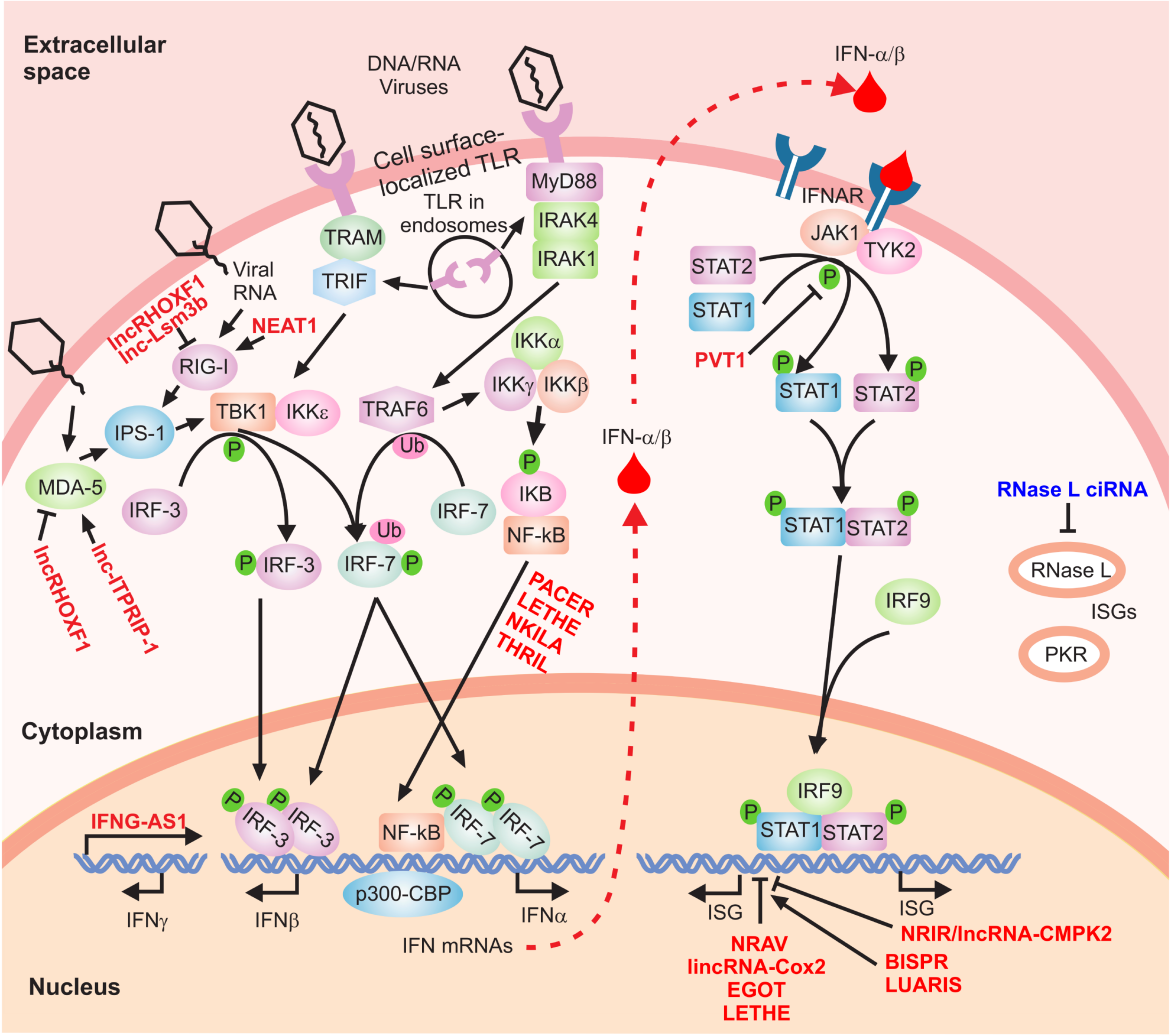
**The IFN signaling cascade.** A simplified scheme of the signaling cascade of type I IFNs is shown. The regulatory lncRNAs are shown in red font. Phosphorylation and ubiquitination events are indicated by small green circles and pink ovals, respectively.

The IFN signaling cascade is initiated through the activation of the IFN genes, which fall into three major categories. Type I IFNs, comprising IFN-α (12 subtypes), -β, -κ, -ε, and -ω, along with IFN-γ, the sole member of type II IFN family, have been the subject of intense studies for decades. The more recently classified type III IFNs include IFN lambda 1, 2, 3, and 4 (IFN-λ1/IL29, IFN-λ2/IL28A, IFN-λ3/IL-28B, and IFN-λ4) [2–6]. The expression of these potent cytokines is induced by a wide range of stimuli. In the case of viral infections, components of the invading microorganisms including foreign RNA and DNA are recognized by a class of molecules that identify pathogen-associated molecular patterns. These include RNA sensors such as RIG-I (retinoic acid inducible gene I), MDA5 (melanoma differentiation-associated gene-5), and TLR (Toll-like receptor) 3, 7, and 8 (Figure 1) [1, 7, 8]. Activation of the sensor molecules leads to initiation of signaling through complex pathways, which ultimately result in transcriptional induction of IFN genes. For example, the presence of viral RNA in the form of short, blunt-ended double-stranded RNA with a 5′-triphosphate end is sensed by the major RNA sensor molecules RIG-I through induction of a conformational change in this protein [9]. This, in turn, leads to the interaction of RIG-I and TRIM25 and induction of downstream signaling, culminating in activation of transcription factors such as IRF3 (IFN regulatory factor 3) that initiate the transcription of the IFN genes (Figure 1) [1, 7, 8]. Similarly, activation of MDA5 leads to a conformational change in the protein and its oligomerization, which in turn leads to interaction with MAVS, followed by activation of TBK1 and IRF3 (Figure 1) [1, 7, 8]. In the case of TLR3, 7, and 8, signaling is mediated through MyD88 and TRIF, followed by phosphorylation and dimerization of IRF3 and 7 and their translocation into the nucleus. Once in the nucleus, they initiate the formation of transcriptional activator complexes in association with other transcription factors such as NF-kB, CBP/p300, and ATF-2/c-Jun (Figure 1) [10–15].

In addition to RNA sensors, cytoplasmic microbial DNA is also detected by a host of DNA sensors including TLR9, AIM2, RNA Polymerase III, IFI16, DHX9, DHX36, DDX41, and DDX60, among others [16, 17]. Some of these DNA sensors share the same downstream signaling molecules as the RNA sensors. For example, TLR9, DHX9, and DHX36 activate the common TLR adaptor MyD88, and a number of other DNA sensors such as DDX41 and IFI16 signal through the STING-TBK1-IRF3 axis [16, 17]. The final outcome of activation of such signaling cascades is transcriptional induction of IFN genes, in addition to other immune cytokines. Type I IFNs are produced by almost all cell types during the antiviral response with the exception of IFN-α, which is mostly expressed in the immune cells including monocytes/macrophages, and the plasmacytoid dendritic cells. IFN-γ is produced by the cells of the immune system including CD4+ and CD8+ T cells, B cells, NK cells, and antigen-presenting cells (APCs). In the case of class III IFNs, although they are expressed in several cell types, their expression is predominantly found in plasmacytoid dendritic cells. Taken together, expression of IFN genes is regulated through activation of a number of signaling pathways which themselves are turned on by a wide range of stimuli, including molecular signatures of invading microorganisms. In addition to the cell type-specific regulation of the expression patterns discussed above, additional regulatory mechanisms control the signaling cascades that activate the IFN genes. For example, the expression of a number of key factors in the pathways described above, including RIG-I and MDA5, is induced by the IFN response, thus creating a positive feed-forward loop that helps perpetuate the IFN response. To allow the termination of the IFN response in a physiologically timely manner, an IFN-mediated feedback mechanism is activated through the IFN-induced protein ISG15, which reduces the cellular level of active RIG-I by directly conjugating to it [18]. In addition to such protein-mediated regulatory loops, recent studies have implicated a new class of regulatory factors, the long non-coding RNAs (lncRNAs), as novel and crucial mediators of such regulatory effects.

## lncRNAS: A UBIQUITOUS, DIVERSE CLASS OF REGULATORY TRANSCRIPTS

Studies in the last decade have firmly established a critical role for lncRNAs in regulation of diverse aspects of cellular function. High throughput transcriptomic studies have proven that a large fraction of the human genome is transcribed into tens of thousands of long transcripts that do not seem to harbor significant peptide-coding capacity [19–22]. These transcripts, named long non-coding RNAs, are thus a heterogeneous class of transcripts and show a wide range of variation in their biogenesis and mechanism of function. They are defined as transcripts that do not code for functional peptides and if they have any impact on cellular function, this impact is mediated through the RNA molecule itself. Interestingly, some transcripts both code for functional peptides and have an independent function as an RNA, and thus fall into the category of bifunctional RNAs [23, 24]. In terms of their length, lncRNAs range from tens of thousands of nucleotides to around 200 nucleotides, with this lower limit being an arbitrary and functionally inconsequential cutoff proposed merely to help distinguish them from the small cellular non-coding RNAs such as snRNAs and snoRNAs [22, 25, 26]. Many lncRNAs are RNA polymerase II transcripts and their biogenesis shows a strong resemblance to that of protein-coding RNAs [27–30]. A large fraction of them are spliced and polyadenylated, although they contain fewer introns and on average have a higher percentage of retained introns compared to protein-coding RNAs [27, 28, 30, 31]. However, lncRNAs are also transcribed by RNA polymerases I and III and a significant subset are not polyadenylated [27–29].

**Figure 2.**
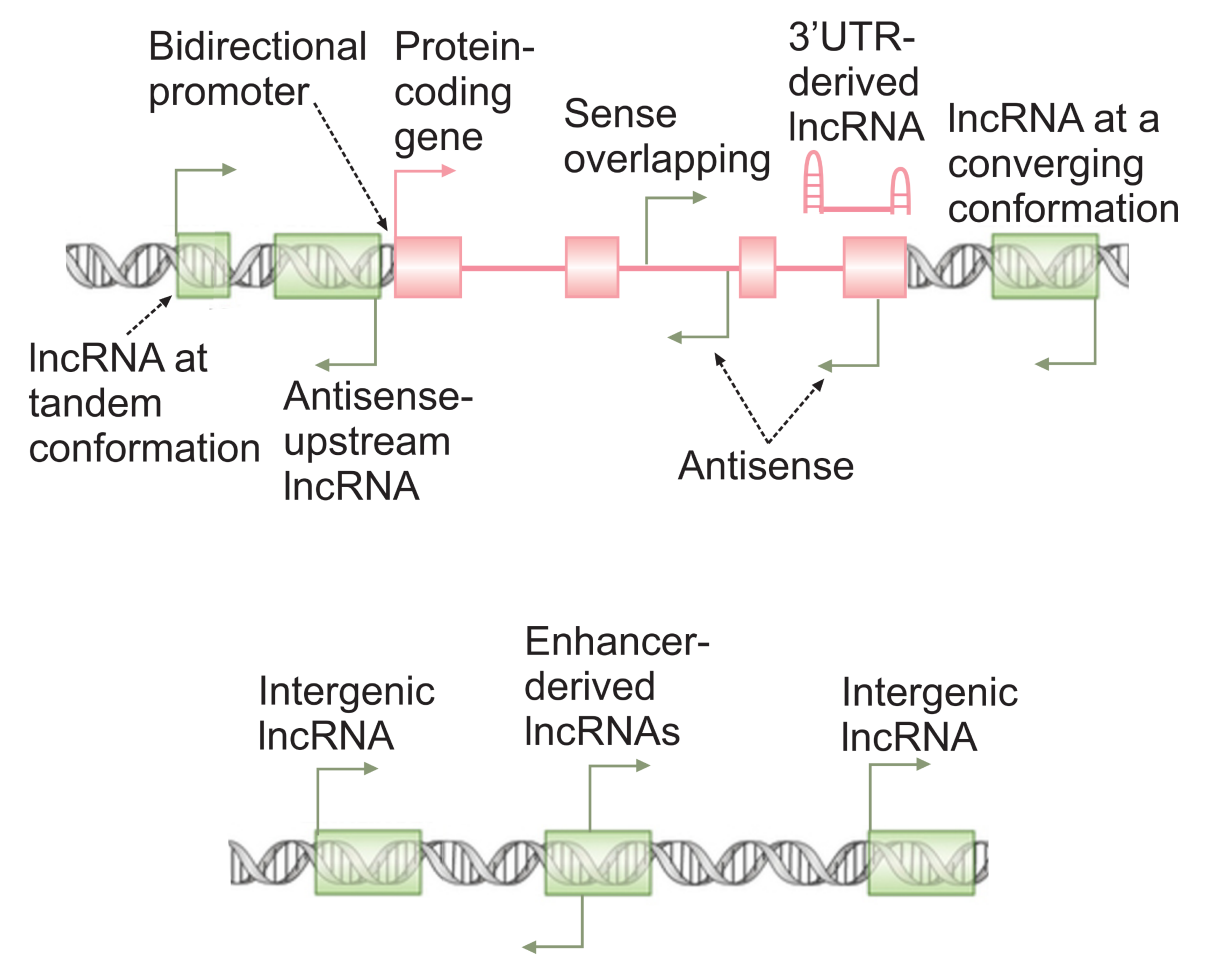
**lncRNAs arise from diverse genomic loci.** The broken arrows mark the location of transcription start sites and direction of transcription. A protein-coding gene is shown in pink, with green rectangles marking the loci of non-overlapping lncRNA genes.

Due to their large number and the recent availability of technologies suited to their study, only a small fraction of lncRNAs have been subjected to ex-silico analysis in vitro or in vivo. However, data obtained from the admittedly limited studied cases point to a ubiquitous and critical role for members of this class of RNAs in cellular function. Both high throughput transcriptomic studies and in vitro and in vivo analyses have shown that, compared to protein-coding genes, the expression of lncRNAs as a group shows a much stronger variation between cell types and at various cellular states within the same type of cell [19, 22, 28, 29, 32]. Indeed, the expression of many lncRNAs is limited to a single cell type, or a single cellular state—for example, after stress or following exposure to a certain cytokine [19, 28, 29, 32]. This interesting characteristic suggests that this class of RNAs may constitute a uniquely promising class of therapeutic targets, as the specificity of their expression limits off-target effects. On the other hand, such a high level of expression specificity points to the presence of a large number of lncRNAs that have not yet been described or annotated because the particular cellular state that induces their expression has not yet been subjected to high throughput sequencing analysis. Consistent with this possibility, increasing the depth and breadth of sequencing studies has almost always led to the discovery of a large number of previously unannotated lncRNAs and it is likely that many identified promoter-like elements in the human genome may in fact give rise to hitherto unknown lncRNAs [33–35]. Further, alternative processing of transcripts originating from protein-coding loci frequently leads to generation of RNAs that lack protein-coding capacity, and thus are non-coding isoforms of protein-coding transcripts (Figure 2) [27, 29, 33]. Thus, the total number of lncRNAs in the human transcriptome and their impact on cellular function is likely to be much higher than is currently thought.

As mentioned above, existing data indicate that lncRNAs are likely to be ubiquitously involved in regulation of nearly every aspect of cellular function [19, 22, 32, 36–39]. High throughput transcriptomic studies have shown that the majority of lncRNAs are predominantly or exclusively localized to the nuclear compartment [27–29]. Consistent with their localization pattern, a large fraction of studied lncRNAs seem to be involved in regulation of nuclear processes, including transcriptional and epigenetic regulation [21, 22, 32, 40]. The mechanism of function of lncRNAs, similar to their mode of biogenesis, is highly heterogeneous. A subset of lncRNAs affect cellular function by virtue of being transcribed, that is to say, through the impact that their transcription has on the chromatin state of their own locus and the vicinity [21, 22, 40]. A large fraction of lncRNAs are located close to or even overlap key protein-coding genes or the enhancers that regulate them (Figure 2) [28, 29]. Large-scale transcriptomic studies have shown that many lncRNAs originate from promoters within an intronic or exonic region of another gene and are transcribed in sense or antisense orientation compared to the gene they overlap (Figure 2) [27–29, 41–43]. It's conceivable that transcription of such overlapping lncRNA genes may impact the chromatin or transcriptional state of the genes around them. Further, bidirectional transcription from the promoter of over 10% of human genes results in two stable transcripts sharing the same promoter, and in many cases at least one of the two promoter-sharing transcripts is a lncRNA (Figure 2) [44–46]. In several studied bidirectionally transcribed loci, active transcription of one promoter-sharing partner either enhances or inhibits the transcription of the other one, and this effect is likely to be at least partly dependent on the act of transcription itself [45, 47]. Another subclass of lncRNAs is transcribed from enhancer loci (Figure 2) and is thought to be critical for the function of enhancers [48–50]. While the exact mechanism through which such enhancer-associated lncRNAs mediate their cellular effect has only been studied in a few examples, it is likely that the act of transcription of such RNAs is a major contributor to enhancer activity [51–56]. However, the enhancer-associated lncRNAs themselves may also be important for the function of at least a subset of enhancers [48].

For many studied lncRNAs, their impact on cellular function is mediated through the transcribed RNA itself. In some cases, the nascent RNA, while it is still being transcribed, interacts with cellular proteins or other cellular RNAs and increases their local concentration, or modulates their function in a way that affects the transcriptional or chromatin state of their own locus or the vicinity [21, 22, 40]. Thus, for a fraction of lncRNAs, their genomic locus may provide clues into their potential targets (Figure 2). However, it should be mentioned that in some studied examples, the nascent lncRNAs affect loci that may even be on a different chromosome, but are located in their proximity and within the same nuclear territory in the three-dimensional structure of chromatin [22, 40, 57, 58].

In contrast to the two lncRNA functional classes described above, many lncRNAs including many of those originating from intergenic regions (Figure 2) impact cellular function through their fully mature transcripts. Such RNAs frequently affect the function of the macromolecules with which they associate. In some studied cases, a lncRNA blocks a functional sequence such as a splice site or an miRNA binding site in another transcript through forming a basepairing interaction with a complementary sequence in the target transcript [42, 59, 60]. Another example for this base-pairing mediated mechanism of function is provided by 3′UTR-derived lncRNAs, which result from selective stabilization of the 3′ ends of protein-coding genes (Figure 2) [61]. Such lncRNAs can act as miRNA sponges, as they can compete for binding to miRNAs that target the original protein-coding transcript from which they were derived. Another very common mechanism of function for lncRNAs is through modulation of the function of proteins that interact with them. This can be achieved by directly affecting the function of a certain macromolecule, for example via molecular mimicry, to competitively inhibit the binding of a protein to its target [22, 40]. Alternatively, a lncRNA may act as a scaffold to bring two macromolecules, such as two proteins or a protein and a certain region of DNA, close to each other, eliciting a certain interaction between them [21, 22, 40]. As mentioned above, lncRNAs seem to play a ubiquitous role in the regulation of almost every aspect of cellular function, and recent studies have indicated their extensive involvement in diverse aspects of immune function, including the IFN response [62–70]. The following sections of this review will focus on the role of the host-derived lncRNAs in the IFN response, although it should be mentioned that additional virally-coded lncRNAs and many additional host-derived lncRNAs also affect the overall antiviral response and thus, indirectly, the IFN response (reviewed in [69, 71, 72]).

## REGULATION OF THE INDUCTION OF THE IFN RESPONSE BY lncRNAS

As discussed above, recognition of pathogen-associated molecular patterns by a diverse set of sensor molecules leads to activation of several signaling cascades, which ultimately lead to transcription of the IFN genes. As this is the decisive step in the launch of the IFN response, it is tightly regulated by a number of known protein-mediated mechanisms [1, 7, 8]. However, emerging data point to a key role for lncRNAs in regulation of the induction of IFN genes (Figure 1). Several lncRNAs play pivotal roles in regulation of the expression or function of critical sensor molecules. An interesting, very recently identified example is lnc-Lsm3b, a non-coding alternatively processed isoform of the protein-coding gene Lsm3 [73]. lnc-Lsm3b is induced in response to extended exposure to higher doses of type I IFN. Once the level of the lnc-Lsm3b accumulates in the cell in the later stages of the IFN response, using structural mimicry, it competes with virally-encoded RNAs for binding to RIG-I and, upon binding, stabilizes the inactive structure of RIG-I [73]. Importantly, both in cultured human cells and in lnc-Lsm3b knockout mice, loss of function of lnc-Lsm3b led to an enhanced IFN response [73]. This is certainly a very attractive model for an RNA-mediated feedback loop, with an IFN-induced lncRNA blocking the action of other RNAs, virally-derived ones that would otherwise perpetuate the IFN response. However, an important question is whether this regulatory mimicry is an exclusive property of this lncRNA, or whether a population of cellular RNAs that meet a certain structural requirement also can induce the same effect. Structural studies on lnc-Lsm3b showed that the critical structural elements for binding to RIG-I in a productive manner included an RNA duplex structure with GA-rich motifs containing asymmetric internal loops [73], which can potentially be found in many cellular RNAs. Surprisingly, a 5′ triphosphate was not needed for the lnc-Lsm3b to bind RIG-I in vitro [73]. Thus, it is plausible that a number of other IFN-induced RNAs also show similar negative feedback activity, either via binding to RIG-I or to other viral RNA sensors in the cell.

Using a closely related mechanism, lnc-ITPRIP-1, which is induced in response to IFN stimulation [74] and infection by both RNA and DNA viruses, positively regulates the function of MDA5, another key cytoplasmic RNA sensor [75]. Through direct association with MDA5, lnc-ITPRIP-1 promotes the conformational changes associated with activation in MDA5, leading to its oligomerization and induction of downstream signaling through IRF3. Studies in hepatoma cell lines indicated that lnc-ITPRIP-1 enhanced HCV-triggered production of IFN-β, IFN-λ1 and IFN-λ2 in a manner that was independent of RIG-I and fully mediated through MDA5 [75]. Unlike lnc-Lsm3b, lnc-ITPRIP-1 seems to enhance or stabilize the binding of at least some viral RNAs to the RNA sensor MDA5 [75], possibly acting as an allosteric regulator.

Additional lncRNA-mediated feedback mechanisms involving sensor molecules have been reported for the lncRNAs NEAT1 and lncRHOXF1 (Figure 2). NEAT1, which forms a key structural component of paraspeckles, is induced in response to infection with the Hantaan virus in human umbilical vein endothelial cells in a IRG-1/IRF7-dependent manner [76]. Increased NEAT1 expression level, in turn, leads to localization of proline- and glutamine-rich protein (SFPQ), a splicing factor and a transcriptional repressor, to paraspeckles. The outcome of this sequestration of SFPQ is removal of its transcriptional inhibitory impact on RIG-I and DDX60 loci, leading to IFN-β production [76]. Thus, unlike lnc-Lsm3b, NEAT1 creates a positive feed forward loop that potentiates IFN production during viral infection. Another lncRNA, lncRHOXF1, is expressed in human embryos at the blastocyst stage in trophectoderm and primitive endoderm and its knockdown leads to the induction of expression of RIG-I and MDA5, and upregulation of IFN-β production [77]. Although the mechanism of this effect is not known, existing data point to a negative regulatory role for lncRHOXF1 in the IFN response.

As mentioned above, some lncRNAs impact the expression of genes that overlap their loci. The human IFNG/IFN-γ locus overlaps that of lncRNA IFNG-AS1 (IFN Gamma AntiSense-1, also known as Tmevpg1 and NeST) [78–81], which is expressed in NK cells in addition to CD4+ and CD8+ T cells. Interestingly, the expression patterns of IFNG-AS1 and IFNG show a strong correlation [78, 79, 91]. IFNG-AS1 expression is required for efficient expression of IFN-γ during CD8+ T cell activation and polarization of Th1 CD4+ cells. Existing data indicate that the mature transcript of IFNG-AS1 interacts with and likely recruits WDR5—which is a subunit of the MLL/ SET1 histone H3 lysine 4 methyltransferase complex—to the IFNG locus, thus activating its expression [79, 80]. Taken together, the few studied examples indicate that lncRNAs exert a strong regulatory effect at multiple steps during the induction of the IFN response. The high throughput screen performed by Jiang et al. for RNAs that bind RIG-I yielded a number of strong hits, only one of which was studied [73]. Analysis of the function of additional RIG-I-interacting RNAs and performing similar high throughput studies for other key factors in the signaling cascades involved in the induction of expression of IFN genes is likely to reveal a highly complex cell type-and context-specific web of regulatory interactions. In addition, in-depth analysis of the results of such high-throughput binding studies, especially in the case of sensor molecules, can help fine-tune our knowledge of the mode of recognition of self and non-self molecular patterns during the induction of IFN response.

## THE IFN CASCADE AND TRANSCRIPTIONAL REGULATION OF PROTEIN-CODING AND NON-CODING TRANSCRIPTOME

As mentioned above, the outcome of the signaling and regulatory interactions discussed above is induction of the expression of IFN genes. Once induced, these potent cytokines act in an auto-crine and paracrine manner by activating their cognate receptors to launch the potent antiviral IFN response. Although the three different categories of IFNs bind distinct receptors, they show extensive overlap in their downstream transcriptional cascades and activate overlapping sets of genes [1, 82–86]. All type I IFNs signal through the dimeric receptor IFNAR, resulting in the induction of the Janus Kinse/Signal Transducers and Activators of Transcription (JAK/STAT) signaling pathway (Figure 1) [1, 7, 8]. This, in turn, results in phosphorylation of STAT1 and STAT2 and their nuclear translocation as a dimer. In a very similar manner, signaling by IFN-γ is mediated through the heterodimeric IFN-γ receptor, leading to subsequent signaling through phosphorylated, dimerized STAT1 [84]. The lambda IFNs bind a receptor complex comprising IL10R2 and IFNLR1 (CRF2-4 and IL28RA, respectively) [6], leading to downstream signaling through the JAK/STAT and MAP Kinase pathways [87]. Thus, while lambda IFNs are structurally similar to the IL-10 family of cytokines, their downstream signaling and antiviral activity resemble that of types I and II IFNs [82, 85, 88]. Once in the nucleus, the homodimeric and heterodimeric STAT complexes interact with additional transcription factors such as IRF9/p48, forming activated transcriptional complexes that induce the expression of hundreds of IFN-stimulated genes (Figure 1) [1, 7, 8].

A number of lncRNA-mediated regulatory mechanisms act on the above signaling cascades to modulate the IFN response. In a recent study, it has been shown that in the context of hepatocellular carcinoma cells, IFN-α stimulation leads to upregulation of expression of lncRNA PVT1, which is known to be involved in several cellular processes including oncogenesis [89]. Knockdown studies indicate that PVT1 interacts with STAT1 and reduces its phosphorylation level, thus blocking the IFN-α signaling pathway as a negative feedback mediator [89]. A similar negative regulatory effect, albeit protein-mediated, is exerted in THP1 cells by STAT3, which is activated by type I IFNs during the IFN response [90–92]. However, the impact of STAT3 on the IFN response is complex and context-dependent. STAT3 can sequester STAT1 and prevents its dimerization in THP1 cells [90], suppress the IFN response directly via its N-terminal domain in MEFs [92], or conversely play an antiviral role in Huh7 and A549 cells where it is needed for the induction of a subset of ISGs [91]. Interestingly, activated STAT3 induces the expression of lncRNA Lethe, which in turn, through an unknown mechanism, blocks the expression of the ISGs PKR, OAS, and IRF1 and promotes the replication of the hepatitis C virus [93]. STAT3 itself is subject to lncRNA-mediated negative regulation through the IFN-γ-induced lncRNA00364 [94]. It has been shown that, in hepatocellular carcinoma cells, lncRNA00364 can specifically bind STAT3 and inhibit its phosphorylation, thus abating its negative transcriptional control and resulting in upregulation of IFIT2, an antiviral ISG [94].

As mentioned above, the ultimate outcome of the IFN response is activation of a strong transcriptional cascade leading to changes in expression of a large number of genes, including both protein-coding and non-coding RNAs. Several ISGs, such as FIT-1, IFIT-2, IFITM3, ISG15, ISG20, RNase L, PKR, RSAD2/Viperin, and BST2/Tetherin are known to have direct or indirect antimicrobial activity through regulation of expression of additional genes [14]. As many IFNs can bind their receptors in both autocrine and paracrine manners, activation of the IFN cascade leads to a cell-intrinsic antimicrobial state, thus limiting the spread of the invading micro-organisms. While most cells are able to launch the type I IFN response, the magnitude of the response and the exact set of genes induced in response to IFN signaling show a strong dependence on cell type and context [1, 7, 8]. Further, an additional layer of control in the form of negative feedback loops mediated by ISGs and other cellular signaling pathways regulate the duration of the response [1, 7, 8, 95, 96]. For example, production of IFN-γ is positively regulated by IL2, IL12, and IL18 and negatively regulated by the IL-4, IL-10, and TGF-β pathways [97]. Similarly, key cellular pathways such as NF-κB, NFAT, JNK, ERK, and p38 MAPK pathways are involved in mediation of the activation of IFN genes. While future studies will reveal additional examples of lncRNA-mediated feedback regulation acting on the core IFN signaling cascade, this class of RNAs likely also mediates a fraction of regulatory loops that coordinate the above-mentioned signaling pathways during the innate immune response. Emerging evidence indicates that key cellular signaling pathways such as NFkB and NFAT, which strongly affect the IFN response, are heavily associated with lncRNA-mediated regulation (for example, see [98–103]). As the expression of lncRNAs is highly cell type- and context-specific, it is also likely that their involvement in such regulatory networks will prove to be at least partially responsible for the cell type-specific aspects of the IFN response.

## THE RESPONSE OF THE LONG NON-CODING TRANSCRIPTOME TO IFNS

While IFN-induced changes in the expression of protein-coding RNAs and their functional outcome have been well documented for over two decades, our knowledge of the impact of IFNs on lncRNA genes is highly incomplete. The first evidence for the involvement of the lncRNAs in the transcriptional response to IFNs was provided by a RNA-seq study in mouse lung tissue and cultured mouse embryonic fibroblasts, which showed that a sizable fraction of the total transcriptional response to treatment with IFN-β or infection with influenza A virus belonged to lncRNA genes [104, 105]. Interestingly, the induction of many lncRNAs was dependent on STAT1 and showed a dynamic, complex pattern following IFN stimulation [104, 105].

The first high throughput studies in human cells, which were performed on primary human hepatocytes from five donors of different ages and genders, largely mirrored the results obtained in the mouse [74]. Not only did a large number of lncRNAs show differential expression following IFN-α stimulation, but they also showed a dynamic expression pattern, with different lncRNAs showing distinct temporal patterns and magnitude of response to IFN stimulation. The differentially expressed lncRNAs included a large number of transcripts that were downregulated in response to IFN treatment, in addition to many upregulated RNAs. Interestingly, analysis of the genomic loci of the differentially expressed lncRNAs indicated that a significant fraction of them were either located in close proximity to or overlapped with protein-coding genes. Many such pairs showed changes in expression in the same or opposite direction after IFN stimulation [74, 106]. Since many lncRNAs seem to regulate the expression of nearby genes, the above observations pointed to the potential presence of lncRNA:protein-coding RNA regulatory loops during the IFN response [74, 106]. Additional studies in human cell lines, including studies focusing on later time points in the IFN response, largely confirmed these conclusions [107, 108]. Importantly, these studies in aggregate identified a large number of potential targets for in-depth study of the role of lncRNAs in the IFN response.

## REGULATION OF THE IFN-MEDIATED TRANSCRIPTIONAL CASCADE BY lncRNAS

Among the IFN-induced lncRNAs identified in the human primary hepatocytes by RNA-seq, one lncRNA, which originated from a locus neighboring the ISG CMPK2, showed the highest level of induction [74]. The IFN-induced upregulation of this lncRNA was mediated by the JAK-STAT signaling pathway and its promoter area contained an ISRE (IFN-Stimulated Response Element), suggesting that it was a bona fide ISG. Functional studies indicated that the lncRNA, which was named lncRNA-CMPK2 or NRIR (Negative Regulator of the IFN Response), negatively regulates several protein-coding ISGs. NRIR's target ISGs included the two ISGs neighboring its locus, CMPK2 and RSAD2/Viperin, along with ISGs transcribed from non-neighboring loci such as ISG15, CXCL10, IFITM1, and IFIT3. The NRIR-induced reduction in ISG expression resulted in an ineffective IFN response and an increase in replication of HCV [74]. These results, combined with the strict nuclear localization of NRIR RNA, suggest a role in transcriptional or epigenetic regulation for NRIR. Interestingly, in addition to hepatocytes, NRIR was induced in diverse cell types in both mouse and human after IFN stimulation [74], suggesting that it may be involved in negative regulation of the IFN response in diverse tissues.

A high-throughput study of genes differentially expressed in response to infection with the H1N1 influenza virus in human alveolar A549 cell line yielded a number of differentially induced lncRNAs, one of which, named NRAV (Negative Regulator of Antiviral Response), was chosen for further study [109]. In addition to influenza virus, the level of NRAV was significantly reduced after infection with additional viruses in multiple cell lines. Gain and loss of function studies in both cultured human cells and transgenic mice overexpressing human NRAV indicated that the level of viral replication shows a direct relationship with the expression level of NRAV. Interestingly, functional studies indicated that NRAV, similar to NRIR [74], negatively regulated the expression of a number of protein-coding ISGs [109]. Unlike NRIR, none of the NRAV-regulated ISGs (IFIT2, IFITM3, IFIT3, OASL, ISG20L2, and MX1) are transcribed from neighboring loci. Interestingly, NRAV is transcribed from what is likely a bidirectional promoter in an opposite direction to the main isoform of the dynein light chain gene DYNLL1. However, changes in NRAV expression did not correlate with DYNLL1 expression [109].

Additional evidence for a negative regulatory role for lncRNAs in IFN response came from the study of lncRNA EGOT (Eosinophil Granule Ontogeny Transcript) [110], which was discovered during a study in eosinophils. EGOT is expressed during the development and maturation of eosinophils, and is thought to regulate key functional genes such as the major basic protein and the eosinophil derived neurotoxin [110]. It overlaps an intron of the inositol 1,4,5-trisphosphate receptor 1 (ITPR1) gene in an antisense orientation; however, changes in EGOT expression did not affect the expression level of ITPR1 mRNA (Prior *et al*., unpublished observation). A high-throughput screen in the Huh7 human hepatocyte cell line for genes differentially expressed after viral infection indicated that EGOT is strongly induced after infection with a number of RNA viruses [111]. Analysis of the mechanism of induction of EGOT indicated that it depends on activation of PKR and to a lesser extent RIG-I RNA sensors, which induce EGOT expression through activation of the NFkB axis [111]. Very high doses of IFN-α also led to induction of expression of EGOT, albeit at much lower levels compared to those observed after HCV infection [111]. Interestingly, loss of function studies indicated that, similar to NRIR and NRAV, EGOT negatively regulated the expression of a number of protein-coding ISGs, including GBP1, ISG15, MX1, BST2, ISG56, IFI6, and IFITM1, and thus reduced the impact of the IFN response on viral replication [111].

Unlike the lncRNAs described above, a recently identified lncRNA is a positive regulator of the IFN response. This lncRNA, named LUARIS (lncRNA upregulator of antiviral response interferon signaling, also lncRNA#32) was identified in a screen for genes that were differentially expressed in an IRF3-dependent manner after poly(I:C) treatment in human immortalized HuS hepatocytes [112]. LUARIS, like EGOT, is an antisense lncRNA and overlaps parts of introns 21 and 22 and the intervening exon of the protein-coding gene HECW1. Expression level of LUARIS showed a decrease after IFN-β stimulation, and loss of function studies in HuS and THP1 cells indicated that the expression level of LUARIS showed a direct correlation with the expression of several ISGs, including IRF7, OASL, RSAD2, CCL5, CXCL11, and IP-10. Further, loss of expression of LUARIS led to increased viral replication during HCV and HBV infections, indicating that expression of LUARIS had a positive regulatory impact on the IFN response [112]. Additional studies suggested that the transcriptional stimulatory effect of LUARIS was likely mediated through its interaction with ATF2 (activating transcription factor 2) [112]. While downregulation of a positive regulator of the IFN response during activation of the IFN cascade is counterintuitive, it is plausible that the expression level of such positive regulators can be adjusted through additional feedback mechanisms in order to fine-tune the magnitude of the expression of their targets and thus, that of the antiviral and inflammatory response. This interesting possibility underscores the need for analysis of additional examples of lncRNAs that show decreased expression in response to IFN stimulation.

One of the most upregulated lncRNAs after IFN stimulation in primary human hepatocytes was an annotated but unstudied transcript originating from the promoter of the well-studied ISG BST2 in the opposite direction to that of the protein-coding transcript [74, 106]. Analysis of this locus indicated the presence of a bidirectional promoter containing an ISRE element, and independent studies by two groups confirmed that this lncRNA, which was named BISPR (BST2 IFN-Stimulated Positive Regulator), was indeed induced after IFN stimulation in diverse cell types in a JAK/STAT-dependent manner [74, 106, 107, 111]. Knockdown of BISPR reduced the magnitude of induction of BST2 in response to IFN treatment, suggesting that the expression of BISPR may have a regulatory impact on its promoter-sharing neighbor [106, 107]. This was confirmed by studies in which forced overexpression of BISPR from a transgene led to the induction of expression of BST2 [106]. Importantly, these studies indicated that the mature transcript of BISPR, rather than the act of transcription of this gene, is responsible for the increase in expression of BST2. Interestingly, the induction of expression of BISPR in response to IFN preceded that of BST2 in several studied cell lines, which suggested that the earlier induction of BISPR helped facilitate the expression of BST2 in response to IFN stimulation [106]. Whether BISPR regulates the expression of any genes other than BST2 is not known. However, since the impact of BISPR on BST2 expression is at least partly mediated through its mature transcript, it is plausible that BISPR RNA may play a similar role in additional loci.

Taken together, studies from a very small number of lncRNAs that comprised the very top hits in high-throughput studies, have indicated a powerful role for this class of transcripts in the IFN response. It is very likely that study of additional candidates from the list of lncRNAs identified by high-throughput studies will strongly expand the known lncRNA-mediated regulatory mechanisms in the IFN response. Three of the four studied lncRNAs with more than one known target act as negative regulators, with partially overlapping target ISGs. Interestingly, a number of proteins with negative regulatory impacts on the IFN response have been previously described [1, 7, 8, 95, 96]. Integration of the known protein-mediated regulatory loops with the emerging lncRNA-mediated ones in future studies will yield a unified picture of the regulatory mechanisms that control the duration and magnitude of the IFN response.

## CONCLUSIONS

Although research in the last decade has revealed a critical role in biology of higher eukaryotes for long non-coding RNAs, study of the impact of this class of RNAs on IFN response is still in its infancy. However, even with the very small number of studied examples, it is clear that lncRNA-mediated regulatory loops constitute a ubiquitous feedback network acting at multiple levels and, in many cases, converging on the same regulatory target molecules with concordant or opposite regulatory effects. This arrangement, in turn, provides the opportunity for extreme fine-tuning of the IFN response to match the character and intensity of the infection and the temporal progression of the inflammatory response. As the lncRNAs studied so far constitute the very top hits identified in a few high throughput studies, it is almost certain that the magnitude of the involvement of lncRNAs in regulation of the IFN response is much higher than currently thought. In-depth analysis of additional candidates from existing lists, and the use of functional high-throughput methods such as loss of function screens are certain to identify a host of additional regulatory lncRNAs in the IFN response, and offer a new perspective not only on the innate immune response against pathogens, but also on pathologies characterized by misregulation of the inflammatory response.
